# Merozoite surface protein-3 alpha as a genetic marker for epidemiologic studies in *Plasmodium vivax*: a cautionary note

**DOI:** 10.1186/1475-2875-12-288

**Published:** 2013-08-21

**Authors:** Benjamin L Rice, Mónica M Acosta, Maria Andreína Pacheco, Ananias A Escalante

**Affiliations:** 1 Center for Evolutionary Medicine and Informatics, The Biodesign Institute, Arizona State University, Tempe, AZ, USA; 2 School of Life Sciences, Arizona State University, Tempe, AZ, USA

**Keywords:** *Plasmodium vivax*, Merozoite surface protein-3 alpha, Molecular markers, Parasite diversity, Population genetics, PCR-RFLP, Recombination

## Abstract

**Background:**

*Plasmodium vivax* is the most widespread of the human malaria parasites in terms of geography, and is thought to present unique challenges to local efforts aimed at control and elimination. Parasite molecular markers can provide much needed data on *P. vivax* populations, but few such markers have been critically evaluated. One marker that has seen extensive use is the gene encoding merozoite surface protein 3-alpha (MSP-3α), a blood-stage antigen known to be highly variable among *P. vivax* isolates. Here, a sample of complete *msp-3α* gene sequences is analysed in order to assess its utility as a molecular marker for epidemiologic investigations.

**Methods:**

Amplification, cloning and sequencing of additional *P. vivax* isolates from different geographic locations, including a set of Venezuelan field isolates (n = 10), yielded a sample of 48 complete *msp*-*3α* coding sequences. Characterization of standard population genetic measures of diversity, phylogenetic analysis, and tests for recombination were performed. This allowed comparisons to patterns inferred from the *in silico* simulation of a polymerase chain reaction restriction fragment length polymorphism (PCR-RFLP) protocol used widely.

**Results:**

The larger sample of MSP-3α diversity revealed incongruence between the observed levels of nucleotide polymorphism, which were high in all populations, and the pattern of PCR-RFLP haplotype diversity. Indeed, PCR-RFLP haplotypes were not informative of a population’s genetic diversity and identical haplotypes could be produced from analogous bands in the commonly used protocol. Evidence of frequent and variable insertion-deletion mutations and recurrent recombination between MSP-3α haplotypes complicated the inference of genetic diversity patterns and reduced the phylogenetic signal.

**Conclusions:**

The genetic diversity of *P. vivax msp-3α* involves intragenic recombination events. Whereas the high genetic diversity of *msp-3α* makes it a promising marker for some epidemiological applications, the ability of *msp-3α* PCR-RFLP analysis to accurately track parasites is limited. Local studies of the circulating alleles are needed before implementing PCR-RFLP approaches. Furthermore, evidence from the global sample analysed here suggests such *msp-3α* PCR-RFLP methods are not suitable for broad geographic studies or tracking parasite populations for an extended period of time.

## Background

**Figure 1:**
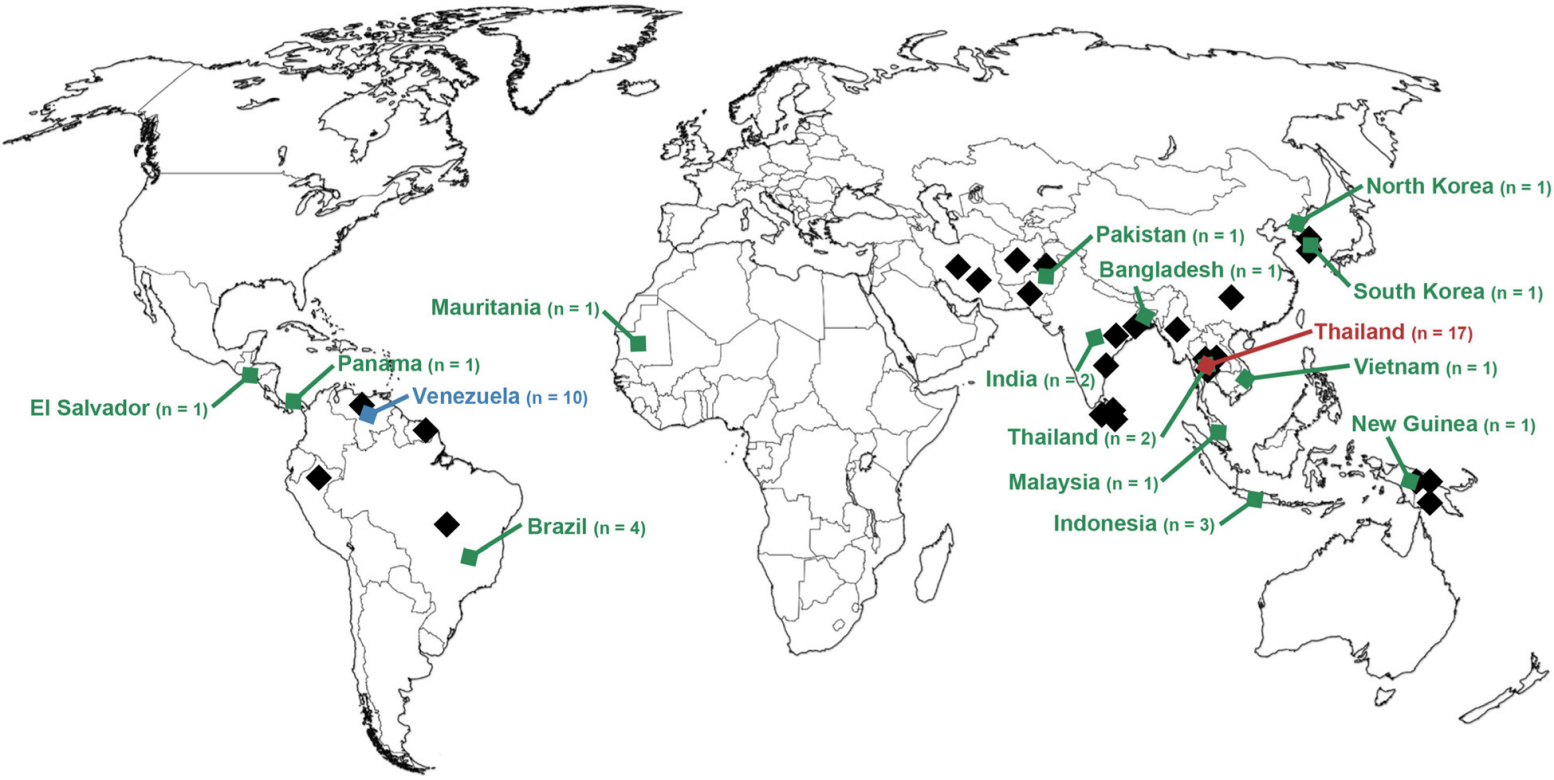
**Geographic origin of the *P. vivax *MSP-3α sequences in this study.** Sampling locations of previously published studies using PCR-RFLP analysis of MSP-3α are marked in black. Sampling locations of the isolates with complete sequences used here are shown in colour.

Though often neglected, *Plasmodium vivax,* with its broad geographic spread, its substantial economic toll, and the billions of people living at risk for its infection, is by itself one of the world’s most burdensome infectious diseases [1,2]. Furthermore, the distinct biology of *P. vivax* is thought to cause additional challenges for malaria control and elimination efforts [3-5]. Due to the need for better *P. vivax* specific epidemiological surveillance tools and the interest in identifying potential vaccine targets, several of the surface proteins identified in *P. vivax* have been extensively investigated [5-7]. Such has been the case for the blood-stage antigen, merozoite surface protein-3 alpha (MSP-3α) [8,9]. Subsequent to finding that the MSP-3α antigen was highly variable in a small initial sample, it was suggested to be a suitable, high resolution marker to distinguish *P. vivax* isolates [10]. As a result, a polymerase chain reaction restriction fragment length polymorphism (PCR-RFLP) assay was developed [10]. Such a protocol has since been used widely; in 24 studies identified, PCR-RFLP analysis of *msp*-*3α* has been used to type 2,000 plus samples from 14 countries across the global distribution of *P. vivax* (Figure 1, see [10-33]). However, beyond the observation that it is highly variable, there have been few studies where MSP-3α polymorphism has been formally investigated [12,16,17].

Evolutionary genetics theory indicates that the interplay of mutations with multiple, different processes, such as natural selection and recombination, can maintain high levels of genetic variability for a locus. For epidemiological investigations focused on tracking and characterizing parasite populations, it is then important to account for what evolutionary forces have shaped the genetic variation at a locus used as a marker. Understanding such processes allows the user to interpret patterns in an epidemiological context. Indeed, two alleles could be similar by convergence due to natural selection (identical by state) but have totally independent origins [34,35]. Thus, rather than just focusing on high diversity *per se*, epidemiologists should consider how evolutionary processes can obscure inferences about population patterns when using specific genetic loci as markers in their investigations.

An important aspect of any technique used in measuring or characterizing genetic diversity is the actual interpretation of the observed polymorphism. Specifically, a critical consideration is whether or not the observation of two identical genetic patterns can be interpreted as evidence that such alleles or genotypes are identical by descent, that is, that they share a common ancestor at a recent time in the past [34]. This allows the user to claim that, in a given epidemiologic context, such identity provides evidence of a relevant event (e.g., recrudescence or separating a local case from an introduction).

To this end, a larger sample set of *msp-3α* sequences was generated in order to better characterize the pattern of genetic variation in *P. vivax* populations at this locus. The ability of a commonly used PCR-RFLP protocol to accurately capture the pattern of *P. vivax msp-3α* diversity was then evaluated. Despite finding substantial nucleotide diversity between isolates, there was limited evidence that variation in the *msp-3α* coding region was structured by geography or by PCR-RFLP haplotype. Rather, diversity was high and similar across geographic regions, and showed a pattern consistent with recurrent recombination. Such complexities are not easily interpretable without suitable sequencing data. This study stresses the importance of locally evaluating the use of PCR-RFLP protocols targeting *msp-3α* as a marker in molecular epidemiologic investigations in *P. vivax*. Furthermore, the problems and limitations observed in *msp-3α* should be considered in other simple PCR based-fragment size genotyping techniques that target repetitive regions in loci encoding merozoite surface antigens.

## Methods

### Parasite sampling

To generate a global sample of the genetic diversity of MSP-3α [PlasmoDB [36] ID: PVX_097720], all publically available sequences were retrieved. In addition, in order to increase the probability of sampling the most divergent *msp-3α* alleles, sequences were newly obtained from ten geographically and temporally diverse laboratory isolates from across the parasite’s broad distribution (Figure 1 and Additional file 1). These strains (with their year of isolation, if available) were: North Korean (1953), Indonesia I (1990), Thai III, Vietnam-Palo Alto (before 1978), India VII (2001), Salvador I (1970), Panama I (1969), Brazil I (1994), Mauritania I (1998), and Chesson from New Guinea (1944). To analyse *msp-3α* diversity at a finer, local scale, sequences of 10 clinical isolates collected in Tumeremo, Venezuela (Bolívar State) in 2003-2004 were also generated. Sequences available in the National Center for Biotechnology Information nucleotide database included 17 sequences of isolates collected from three provinces of Thailand in 2001-2002 by Mascorro et al [16] that allowed assessment of local diversity in another region (accession codes for the *P. vivax* sequences used are shown in Additional file 1). Combined, a sample of 48 complete *P. vivax msp-3α* coding sequences was analysed.

To further understand the evolution of the *msp-3α* gene and its standing diversity, its ortholog in the macaque parasite *Plasmodium cynomolgi*, the most closely related species to *P. vivax* known, was amplified, cloned and sequenced from nine strains. The *P. cynomolgi* samples spanned the species’ South East Asian distribution and included isolates from Peninsular Malaysia (Berok, B Strain, PT1, PT2, Gombak, and Mulligan), Cambodia (Cambodian), Sri Lanka (*P. c. ceylonensis*), and Burma (RO). Generating population polymorphism data for the gene’s ortholog in a closely related species allows more sophisticated tests of natural selection and the ability to observe the pattern of nucleotide variation in a lineage of the gene unbiased by *P. vivax* specific demographic events.

### Sequencing

Due to the previously known variance in gene length among members of the MSP-3 family of *P. vivax* (ranging from 1,100 to 3,700 bp) and among MSP-3α alleles, amplification by PCR was performed using two different enzymes in order to increase the probability of amplifying *msp-3α* alleles that potentially differed in length: AmpliTaq Gold (Applied Biosytems, Roche, USA) and the *TaKaRa LA Taq* (TaKaRa Mirus Bio Inc, Shiga, Japan) known to better amplify longer products. There were two or more independent reactions per sample. 50 μL PCR reactions included approximately 20 ng of total genomic DNA, 1X PCR buffer, 0.05-0.1 mM of each dNTP, and 0.2-0.4 μM of each primer. Other reagent concentrations and the primers used depended on the polymerase used: *TaKaRa* reactions included 2.5 U/μM polymerase and primer sequences of 5’ AAG AAA ATT TAC TYS AAA GGS AGT TAA CCG 3’ and 5’ TGT TCT CAA YCG ACA TGC RAA TTR GCT AG 3’; for AmpliTaq Gold reactions, 2.5 mM MgCl_2_ and 0.03 U/μM of polymerase was used with the primers 5’ ATG AAA CAC ACC CGC AGC GTC 3’ and 5’ GCT CAA AAA TAG GTG ATT CAT ATC GG 3’. The *TaKaRa* thermocycle protocol was: an initial denaturation step at 94°C for 1 minute; 35 cycles of denaturation at 94°C for 30 seconds followed by annealing and elongation at 65°C for 3 minutes; and a final elongation step of 10 minutes at 72°C. The AmpliTaq Gold protocol was: an initial denaturation step at 94°C for 4 minutes; 35 cycles of denaturation (94°C for 1 minute), annealing (54-60°C for 1 minute), and elongation (72°C for 2 minutes); and a final elongation step of 10 minutes at 72°C. PCR products were excised from 0.9% agarose gels and purified using the QIAquick DNA gel extraction kit (QIAgen™, Hilden, Germany), then cloned using the pGEM®-T Easy Vector System (Promega, WI, USA). Both strands for multiple clones were sequenced using an Applied Biosystems 3730 capillary sequencer. Sequences generated in this study have the NCBI accession codes: [GenBank: KC935422-KC935447].

### Evolutionary genetic analyses

In order to calculate genetic diversity and infer phylogenetic relationships, three alignments were constructed: *P. vivax* and *P. cynomolgi* species-specific alignments and an interspecies alignment of both *P. vivax* and *P. cynomolgi.* Alignment was performed using the MUSCLE algorithm [37] in SeaView4 [38] on translated sequences, followed by manual editing. Phylogenetic relationships were estimated using Bayesian methods as implemented in MrBayes v3.1.2 [39] and using the neighbor joining (NJ) method [40] implemented in MEGA5 v5.05 [41]. The distance based NJ tree was constructed using the Kimura 2-parameter model of nucleotide substitution to calculate a pairwise nucleotide distance matrix, with invariant rates among sites and 1000 bootstrap replicates to assess the confidence of clades. The pairwise distance matrix was also used separately to identify pairs of more similar sequences.

For the Bayesian methods, a general time reversible + gamma + evolutionarily invariable (GTR + G + I) model was used. This model was the one with the fewest parameters to best fit the data as estimated by MEGA5. Bayesian support for nodes was inferred in MrBayes using 42 x 10^6^ Markov Chain Monte Carlo (MCMC) steps, with sampling every 100 generations. 50% of the samples were discarded as burn-in. Convergence is reached after the average standard deviation of the posterior probability is below 0.01 and the value of the potential scale reduction factor is between 1.00 and 1.02 [39].

Genetic diversity among isolates was estimated using the average number of pairwise nucleotide differences per site (overall mean distance, *d*) in MEGA5. The Jukes-Cantor model of nucleotide substitution was used to correct for multiple mutations when calculating distance, and 1000 bootstrap replicates determined the standard error. To investigate possible signatures of selection, the diversity at synonymous (*dS*) and nonsynonymous (*dN*) sites was calculated separately using the Nei-Gojobori method [42] with the Jukes-Cantor correction. To determine whether the estimated difference between *dS* and *dN* deviated significantly from the null expectation, the standard error of *dS* and *dN* was calculated with 1000 bootstrap replicates and tested using a two-tailed Z test [43], a commonly used evolutionary genetic test (e.g., [44-48]). A null hypothesis of neutrality was assumed, with the expectation that synonymous and nonsynonymous positions would have similar levels of diversity (*dS* = *dN*). Genetic differentiation between subpopulations was estimated using the F_ST_ statistic [49] as calculated in DnaSP v5 [50]. Two further tests of neutrality were performed in DnaSP, Tajima’s test based on the frequency of variants as summarized by the *D* statistic [51], and the McDonald and Kreitman test [52] comparing polymorphism and divergence in the *P. vivax* and *P. cynomolgi* alignment.

To identify putative recombination events, the RDP algorithm [53] within the RDP3 program [54] was used to perform an automated screen of the *P. vivax* alignment. Default parameters for the detection of recombination break points and donor-recipient pairs were used with a significance cut-off of 0.05. Only the events where the donor and recipient sequences could be unambiguously identified were recorded.

### Simulated PCR-RFLP analysis

The PCR-RFLP analysis of Bruce et al [10] that has been frequently used to genotype *P. vivax* clinical isolates for MSP-3α was simulated in order to compare PCR-RFLP haplotype diversity to the pattern of nucleotide diversity inferred from the set of full length sequences. The protocol is described in Bruce et al [10]. Briefly, *msp-3α* genotypes are distinguished by variation in gene length due to insertion-deletion (further referred to as indels) mutations and by sequence variability at restriction enzyme cut sites. For the first step, alleles are separated using variation in the size of the product from amplifying the indel rich region of the gene by nested PCR. Sequences of similar length are then further differentiated on the basis of the banding patterns resulting from digestion of the nested PCR product separately with two restriction enzymes (*Alu I* and *Hha I*).

**Figure 2:**
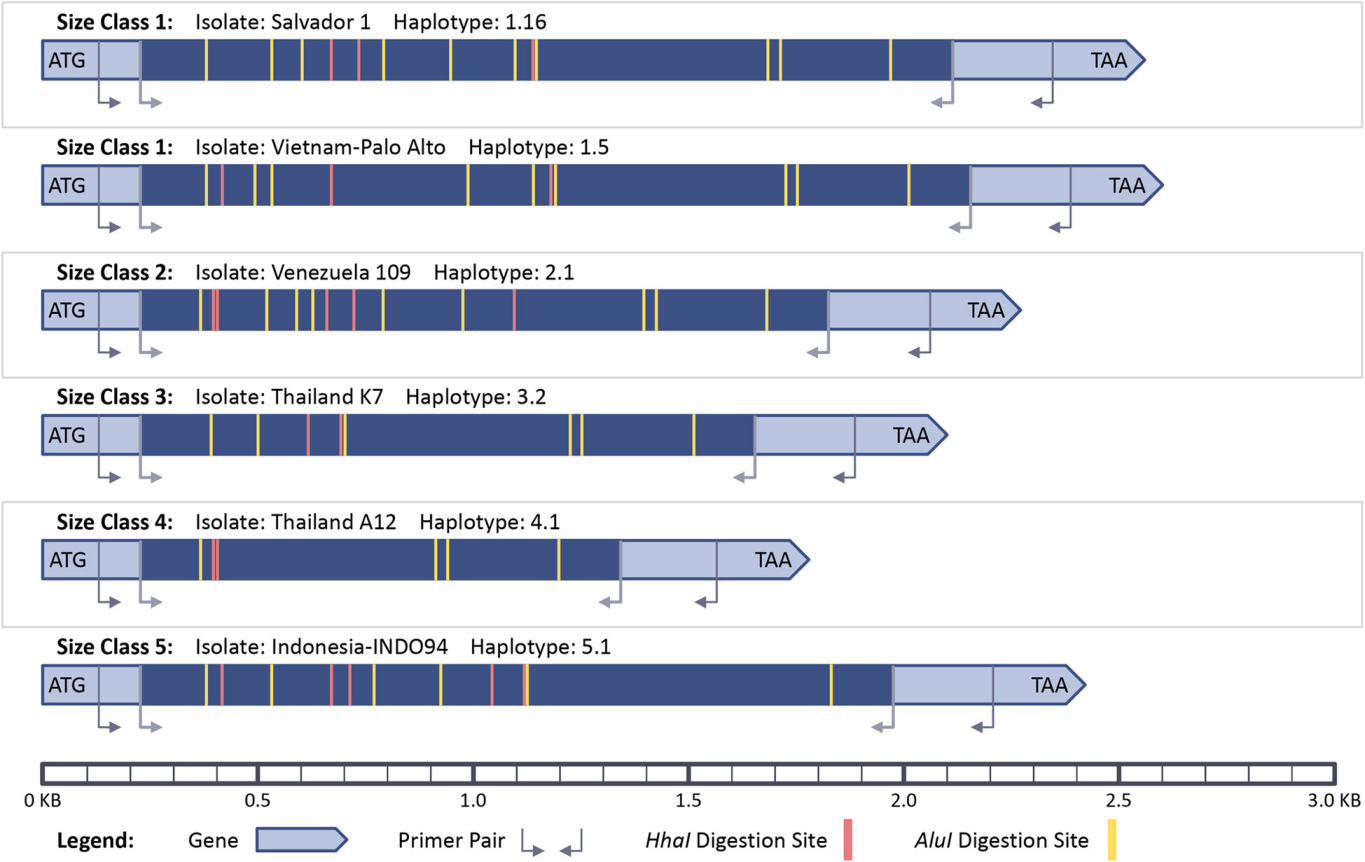
**Schematic of MSP-3α PCR-RFLP protocol for *in silico *digestion.** Drawn approximately to scale, an example from each size class is shown.

To explore this protocol, the nested PCR amplification was simulated *in silico* for the 48 *P. vivax* sequences (see Figure 2). The size of the product expected from amplification with the nested primers was used to organize *P. vivax* sequences into ‘size classes’. Eight of the 48 samples had a single base pair substitution from the nested primers used by Bruce et al [10]. The substitutions’ effects on primer binding are unknown and successful amplification was assumed, allowing all 48 sequences to be assigned to size classes.

*In silico* digestion was then performed on the sequences using a Perl script (modified from [55]). The positions of enzyme cute sites were recorded for both enzymes for each sequence and the resultant restriction digestion bands were arranged by size (Figure 2). To be conservative and to identify the most PCR-RFLP haplotypes possible, it was assumed that only bands differing by less than ±30 bp would be indistinguishable during electrophoresis, a very fine scale detection limit for agarose gels with a standard DNA ladder (Life Technologies™, Carlsbad, CA manufacturer’s protocol). Unique combinations of *Alu I* and *Hha I* digestion bands for samples within a size class were used to create PCR-RFLP haplotypes. Each sequence within a size class was assigned to the corresponding haplotype. As per the Bruce *et al.* protocol, bands less than 100 bp in length were disregarded when determining haplotypes.

## Results and discussion

### Genetic diversity of *P. vivax*

Sequencing and analysis of an augmented sample of *P. vivax* isolates resulted in identifying substantial indel and coding sequence variation between isolates for the gene encoding MSP-3α (see Table 1). Due to indels ranging from 9 to over 800 bp in length, total gene length varied from 1779 (Thai isolate A12) to 2601 bp (strain Vietnam-Palo Alto). As a consequence, the number of informative, alignable sites for determining the genetic distance and diversity among isolates was much less than the gene length for most isolates (Table 1). As observed before (e.g., [10,18]), there were numerous sequences with large (over 300 bp) deletion mutations relative to the Salvador-1 reference strain in the N-terminal half of the gene. However, rather than being due to a single, shared deletion event, such shorter alleles were the result of multiple combinations of indels. Indeed, 20 of the 48 sequences had putative deletions of greater than 300 bp, but among these 20 isolates there were 10 different combinations of indel events. Numerous indels of smaller size were segregating among the sequences, resulting in a total of 28 different indel haplotypes, with an average length of 67.3 bp per indel event, in the total *P. vivax* sample (Table 1).

**Table 1:** **Genetic polymorphism of MSP-3α in *P. vivax *and *P. cynomolgi *populations**

**Species (Population)**	**n**	**Allele range**	**Aligned length**^**b**^	**Inform. sites**	**Indel hap.**	**Average indel length**	***d *(SE)**^**c**^	***dS*-*dN *(SE)**	**Z-stat p value**
*P. vivax*	48	1779-2601	2646	1413	28	67.3	0.027 (0.003)	**+0.021 (0.007)**	*
Worldwide^a^	21	1788-2601	2646	1572	15	46.3	0.027 (0.003)	**+0.025 (0.008)**	*
Thailand	17	1779-2580	2646	1686	13	100.8	0.033 (0.002)	**+0.024 (0.007)**	*
Venezuela	10	2232-2295	2646	2214	4	24.8	0.031 (0.002)	+0.010 (0.005)	-
*P. cynomolgi*	9	1959-1968	2028	1902	6	21.5	0.064 (0.004)	**+0.024 (0.010)**	*

“Inform. sites” stands for “Informative sites” and “Indel Hap.” stands for “Indel Haplotypes”.

“*” Indicates that the value is statistically significant for α = 0.05; “-” stands for non-significant.

^a^ The worldwide population of *P. vivax* contains the geographically diverse samples (n = 21) not within the Venezuelan or Thai sample populations from 2003-2004 or 2001-2002, respectively.

^b^ Lengths and allele range are in base pairs.

^c^ Diversity, *d,* is calculated as the average pairwise divergence per site. Standard error (SE) estimates from 1000 bootstrap pseudoreplications and p-values from the codon based Z test are shown. Significant values (p < 0.05) are bolded.

Sequence polymorphism between isolates varied, but on average there were a high number of segregating (polymorphic) nucleotide sites between isolates in pairwise comparisons (Table 1). Indeed, all *P. vivax* isolates sampled here could be distinguished by their full length gene sequence. The number of sites differing between isolates ranged from a single missense mutation resulting in an isoleucine to phenylalanine change at residue 74 for two Venezuelan isolates, to over 100 segregating sites for multiple pairs of isolates. The average number of nucleotide differences between sequences in the sample was 38.0, for an average genetic distance of 0.027 ± 0.003 differences per site (Table 1). Diversity was not distributed uniformly in the gene, however, with polymorphism at synonymous sites (*dS* = 0.044 ± 0.007) significantly higher than at nonsynonymous sites (*dN* = 0.023 ± 0.003, p = 0.004). This indicates that these aligned sites could be evolving under functional constraints as evidenced by the significant excess of synonymous changes in almost all comparisons from the two-tailed Z test (Table 1).

Diversity among the Thai (*d* = 0.033 ± 0.002) and Venezuelan (*d* = 0.031 ± 0.002) clinical isolates was slightly higher but approximated that seen among the 21 geographically and temporally diverse ‘worldwide’ sample of isolates (*d* = 0.027 ± 0.003). Combining the 27 clinical isolates from these two distant subpopulations with the other 21 diverse isolates did not result in a change in the amount of genetic diversity captured, as average pairwise divergence for the total sample of 48 complete *P. vivax msp-3α* sequences remained 0.027 ± 0.003 (Table 1).

Next, to investigate the relationship between indel and sequence polymorphism, the genetic diversity among the seven indel haplotypes that were shared by more than one sequence was calculated (see Table 2). This comparison is solely phenetic, since homology cannot be assumed in indels patterns. Evidence that these indels are indeed convergences can be found in the fact that sequences sharing the same indels often had distant geographic origins and could have a higher level of nucleotide polymorphism among themselves (*d* = 0.007 ± 0.001 to 0.060 ± 0.005) than the average amount of divergence between any two sequences in the global sample (*d* = 0.027 ± 0.003). This could be partly explained by higher polymorphism at sites within the indel event regions of the gene, as diversity was reduced in the global sample where only the 1413 sites shared by all *P. vivax* isolates were considered (Table 2). However, it is noteworthy that alleles sharing the same indel haplotype could be more similar to sequences with different indel patterns than they were to each other within the same indel haplotype category at the 1413 comparable sites. An example is strain Thai III (indel haplotype 1), which had only two point mutations relative to Venezuelan isolate 168 (indel haplotype 7), but had five relative to the Chesson strain also with indel haplotype 1. This would indicate either that similar indel or point mutations are repeatedly recurring on different backgrounds or that pre-existing variation is being re-shuffled among and between indel haplotypes.

**Table 2:** Genetic diversity among indel haplotypes for MSP-3α

**Indel haplotype**	**Gene length (bp)**	**n**	**Average pairwise divergence per site (SE)**		**Geographic origin of isolates**
			**At all informative sites among the group**	**At sites shared by all isolates**	
1	1788	4	0.013 (0.002)	0.014 (0.002)	New Guinea, Thailand
2	2100	2	0.019 (0.003)	0.009 (0.002)	Thailand
3	2559	5	0.033 (0.003)	0.022 (0.003)	Malaysia, El Salvador, Thailand
4	2550	2	0.060 (0.005)	0.025 (0.004)	Indonesia, Thailand
5	2538	5	0.040 (0.003)	0.026 (0.003)	Mauritania, Bangladesh, Brazil, Thailand
6	2577	2	0.013 (0.002)	0.016 (0.003)	Panama, Thailand
7	2271	7	0.007 (0.001)	0.004 (0.001)	Venezuela

Nucleotide diversity and its standard error (SE) among the 7 indel haplotypes exhibited by more than one *P. vivax* sample (n) is shown.

Another observation was that seven of the 10 Venezuelan clinical isolates constituted indel haplotype 7 and had a substantially reduced level of nucleotide diversity (*d* = 0.007 ± 0.001) (Table 2) relative to the entire *P. vivax* sample. This explains the reduced indel haplotype diversity and reduced average indel size seen in the Venezuelan population (Table 1). It is worth noting, however, that despite this cluster of seven similar sequences, the other three Venezuelan sequences were highly divergent. Their inclusion raised the average pairwise divergence within Venezuela to a value approximately equivalent to that of the diverse global sample (Table 1).

With this evidence that local genetic diversity in both Thailand and Venezuela recapitulated the level of global MSP-3α diversity, a standard measure of population differentiation, the F_ST_ statistic, was used to investigate whether variation at this locus was indeed distributed randomly across populations. The F_ST_ value, which compares the amount of diversity within populations to the amount of divergence between, for the Venezuelan and Thai sample populations was moderately high likely due to the geographic distance (F_ST_ = 0.118).

### Genetic diversity of *P. cynomolgi*

*Plasmodium cynomolgi* is the closest known relative of *P. vivax*[56-58] and it parasitizes simian hosts, mostly of the genus *Macaca*, in Southeast Asia [56]. Sequencing of its *msp-3α* ortholog from nine strains revealed extensive nucleotide polymorphism between strains (*d* = 0.064 ± 0.004), similar to that seen for *P. vivax* (Table 1). Although the average number of segregating sites among the nine *P. cynomolgi* sequences was higher than that observed for *P. vivax*, there was much less indel diversity among the *P. cynomolgi* strains sampled. The difference between the smallest and largest alleles was only 9 bp as a result, as opposed to a difference of more than 800 bp between some *P. vivax* isolates. Maximum gene length for *P. cynomolgi* (1,968 bp) was substantially shorter than for *P. vivax* (2,601 bp). This observation suggests that *P. vivax* may have inherited a shorter gene from its common ancestor with *P. cynomolgi*. In such a case, rather than deletions being responsible for the shorter alleles seen in *P. vivax*, large insertions in a shorter ancestral sequence would explain the range in allele length. The direction of indel mutations cannot be inferred from alignment, however, and the larger *P. vivax* alleles have been found repeatedly to be the predominant type (e.g., [13,26,30,31,33], as was the case in this sample here. Further, diversity among indel haplotypes for sequences with lengths over 2500 bp (indel haplotypes 3-6) varied, but was within the range seen for all haplotype groups in *P. vivax* (Table 2). Diversity would be expected to be reduced among more recently arisen allele types, but this failed to be the case for either the longer or shorter alleles. Finally, due to the prevalence of indel mutations of varying lengths, gene length varied almost continuously and thus it was not possible to easily identify distinct allele ‘types’ based solely on size in most cases.

As with *P. vivax*, a significant majority of the diversity seen in *P. cynomolgi* was constrained to synonymous sites (p = 0.020, Table 1). It is noteworthy, though, that despite it being significantly less than at synonymous sites, diversity at non-synonymous sites in both *P. vivax* (*dN* = 0.023 ± 0.003) and *P. cynomolgi* (*dN* = 0.058 ± 0.005) was substantial, with amino acid changes accordingly frequent. Intraspecies diversity for MSP-3α was comparable to that seen for other merozoite surface proteins, such as MSP-5 [59], and the 200 L fragment of MSP-1 [60], but was substantially higher than for those thought to be evolving under purifying selection such as MSP-7 [61], MSP-8, and MSP-10 [44].

To further test for evidence of a distribution of genetic variation that deviated from expectations of neutrality and to take advantage of the newly generated set of *P. cynomolgi* sequences, the test of McDonald and Kreitman was performed using the *P. vivax* and *P. cynomolgi* alignment. Although there were substantially more non-synonymous than synonymous differences fixed between species, the ratio of fixed to polymorphic sites did not differ significantly (Fisher’s exact test, p = 0.80), indicating that the observed patterns of divergence were still consistent with neutrality. Tajima’s tests of neutrality for both *P. vivax* and *P. cynomolgi* (D = -0.987 and D = 0.392, respectively) were insignificant as well.

### PCR-RFLP haplotype diversity

*In silico* simulation of the initial nested PCR step in the PCR-RFLP protocol of Bruce et al [10] resulted in the identification of five different size alleles in the sample of 48 *P. vivax* sequences (Figure 2). The groups of sequences sharing similarly sized amplicons were termed size classes, and the number of sequences within each varied from 27 in class 1 to just a single isolate (Indonesia-INDO94) in class 5 (Table 3). The expected length of the internal region amplified by nested PCR ranged from 1137 to 1950 bp, consistent with what has been seen previously in locations such as Sri Lanka [32] and Brazil [30]. Alleles with an even larger deletion in the central region of the gene, and thus a smaller amplicon, have been observed in India [26] and Pakistan [24], but none were recovered here. All ten Venezuelan isolates had a predicted nested PCR band size of 1590-1644 bp (gene length: 2232-2295) and were thus in the same class, class 2 (Table 3). However, the geographic origin of the isolates within size classes 1, 3, and 4 (amplicon lengths of 1884-1950, 1449-1461, and 1137-1149 bp respectively) was varied.

**Table 3:** Genetic diversity among PCR size classes and PCR-RFLP haplotypes for MSP-3α

**PCR-RFLP group**	**Band size (bp)**	**n**	**Average pairwise divergence per site (SE)**		**Geographic origin of isolates**
			**At all informative sites among the group**	**At sites shared by all isolates**	
Size Class 1	1884-1950	27	0.052 (0.003)	0.029 (0.003)	Asia, Africa, South America^a^
Size Class 2	1590-1644	10	0.031 (0.002)	0.016 (0.002)	Venezuela
Size Class 3	1449-1461	4	0.029 (0.002)	0.023 (0.003)	Brazil, Thailand, Malaysia
Size Class 4	1137-1149	6	0.024 (0.002)	0.024 (0.003)	Thailand, New Guinea
Size Class 5	1770	1	-	-	Indonesia
Haplotype 1.1	1896-1917	4	0.043 (0.003)	0.025 (0.003)	Korea, Mauritania, Thailand, Brazil
Haplotype 1.2	1896-1920	3	0.048 (0.003)	0.030 (0.004)	Thailand, Brazil
Haplotype 1.5	1929-1950	2	0.033 (0.003)	0.022 (0.004)	Vietnam, India
Haplotype 1.8	1908-1926	2	0.058 (0.005)	0.031 (0.005)	Panama, Indonesia
Haplotype 1.17	1884-1887	2	0.017 (0.002)	0.009 (0.002)	Thailand, Brazil
Haplotype 2.1	1620	5	0.002 (0.001)	0.002 (0.001)	Venezuela
Haplotype 3.2	2100	2	0.019 (0.003)	0.009 (0.003)	Thailand
Haplotype 4.1	1137	5	0.016 (0.002)	0.017 (0.003)	Thailand, New Guinea

Nucleotide diversity for the 5 PCR band sizes and the 8 PCR-RFLP haplotypes with more than one sample (n) is shown. Due to the size differences between alleles, the number of informative sites differs between analyses; both diversity as calculated for all informative sites within the group and only at the sites shared by all isolates in the total sample population (n = 48, number of informative sites = 1413) are shown for comparison. ^a^ Only the continents for the 27 sequences in PCR amplification size class 1 are listed.

*In silico* digestion of the predicted nested PCR products with *Hha I* resulted in *msp-3α* sequences being cut into three to seven bands (Figure 2). Variance among sequences in the size and combinations of these bands, due to sequence diversity at and between cut sites, allowed distinguishing 13 *Hha I* haplotypes among the 27 sequences in size class 1. Three, two and two different *Hha I* haplotypes were identified among sequences in size classes 2, 3, and 4, respectively. Sequences also had from 4 to 13 digestion sites for *Alu I* that resulted in banding patterns that allowed the further discrimination of 10 more *msp-3α* genotypes, for a total of 31 distinct PCR-RFLP haplotypes from the sample of 48 sequences. As a result, 17 of the 48 sequences were within one of the eight PCR-RFLP haplotypes that was shared by more than one sequence after *in silico* digestion with both enzymes. This therefore allowed the genetic diversity within these eight haplotypes to be calculated (Table 3). PCR-RFLP analysis, as simulated here, was unable to resolve all samples to distinct genotypes, and in six of the eight PCR-RFLP haplotypes shared by more than one sequence, the sequences within that group originated from distant geographic locations (Table 3).

An initial observation made when comparing these PCR-RFLP alleles to the pattern of nucleotide diversity was that similarly sized amplicons, the basis of distinguishing PCR-RFLP size classes, were often produced by different indel haplotypes. From 27 sequences with gene lengths of 2526-2601 bp (resulting in an 1884-1950 bp amplicon for digestion using the Bruce *et al.* method) 17 different combinations of indels were observed from sequencing, with indel events averaging 27 bp in length. With numerous indels that averaged a size close to the assumed detection limit of 30 bp segregating into many combinations for class 1, raw amplicon length in many cases simply reflected a convergent sum over these mutation events (indels) and failed to reflect the high levels of MSP-3α polymorphism even within this class. Further, as shown in Table 3, nucleotide diversity within PCR-RFLP size classes (*d* = 0.024 ± 0.002 to 0.052 ± 0.003) varied, but approximated the amount of diversity seen in the total sample population of globally diverse isolates (*d* = 0.027 ± 0.003, see Table 1).

All ten Venezuelan MSP-3α isolates fell within the same size class, and included a cluster of 5 isolates (haplotype 2.1) that had markedly less nucleotide diversity (*d* = 0.002 ± 0.001) than the other PCR-RFLP alleles found in 2 or more samples (*d* = 0.016 ± 0.002 and 0.058 ± 0.005). However, this reduced PCR-RFLP allelic diversity in Venezuela contradicts that the total nucleotide diversity retained within the Venezuelan sample population was similar to that of Thailand, and the entire sample combined (Table 1), despite that those two groups had much greater allelic diversity. More evidence that MSP-3α genetic variation was not structured by PCR-RFLP haplotypes was that isolates with identical banding patterns, such as PCR-RFLP haplotype 1.1 (Table 3), could again exhibit more average pairwise divergence (*d* = 0.043 ± 0.003) than was seen on average between sequences in the entire sample (*d* = 0.027 ± 0.003, Table 1).

**Figure 3:**
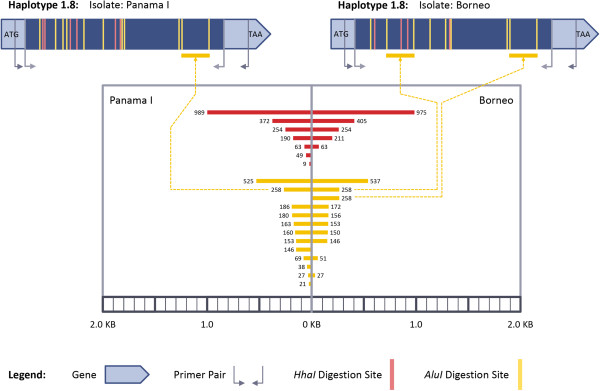
**PCR-RFLP Haplotype 1.8 results from the convergence of analogous bands.** Drawn approximately to scale.

Additionally, bands of similar size, especially in the simulated *Alu I* digestion, were not always homologous. As an example, an isolate from Borneo ([GenBank: AY118174]) [12] and the Panama I strain ([GenBank: KC935447]) had an identical PCR-RFLP haplotype (haplotype 1.8), but converged to an indistinguishable banding pattern through similar sized, but non-homologous bands (Figure 3). From *Alu I* digestion, the isolate from Borneo had two bands of 258 bp, each coming from differing regions of the *msp-3α* gene, while the sequence of Panama I had a single band at that size (Figure 3). In turn, the Panama I isolate had six different bands between 146 and 186 bp in length. Such bands would migrate similarly during electrophoresis and thus the two highly divergent sequences could not be distinguished. Indeed, the amount of pairwise difference between these two sequences with the same PCR-RFLP haplotype (*d* = 0.058 ± 0.005) was almost twice that of the average amount of difference between two sequences in the sample. As a result, PCR-RFLP analysis would have failed to reflect the high level of genetic divergence between the isolates, a subsequent example of identity by state, but not by descent.

The *Alu I* digestion for 47 of the 48 samples resulted in sequences having two or more different digestion fragments from different regions of the gene that differed by less than the ±30 bp discrimination limit. In three additional cases to the one above, identical *Alu I* haplotypes would have been inferred erroneously after electrophoresis due to coincident size in non-homologous bands for divergent sequences. Multiple digestion bands coinciding to have a similar size resulted from the *Hha I* digestion much less frequently, only occurring for six of the 48 isolates; however, similar *Hha I* banding patterns also often failed to correlate to similarity at the nucleotide sequence level. For example, the average pairwise diversity for three *Hha I* RFLP haplotypes (haplotypes H1.1, H1.4, and H2.2) was 0.046 ± 0.003, 0.056 ± 0.005, and 0.064 ± 0.005, respectively, all values much greater than was seen on average between sequences chosen randomly (*d* = 0.027 ± 0.003).

### Phylogenetic analysis and recombination

**Figure 4:**
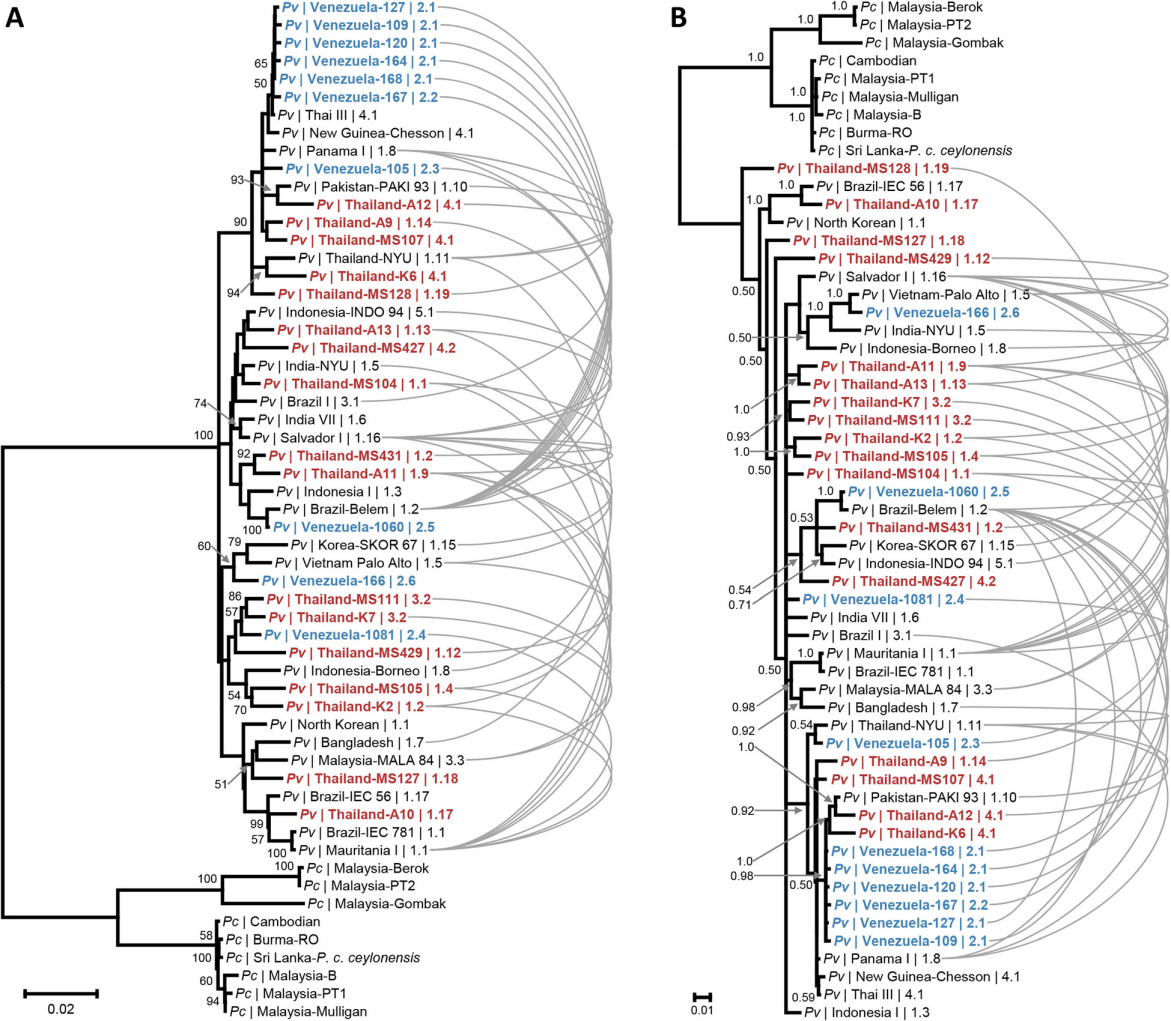
**Phylogenetic tree of *P. vivax *and *P. cynomolgi *MSP-3α sequences.** Trees constructed from Neighbor-joining **(A)** and Bayesian methods **(B)** are shown with nodes with greater than 50% bootstrap or 0.50 posterior probability support, respectively, noted. Sequences are labelled by species (*Pv* or *Pc*), country of isolate origin, and PCR-RFLP haplotype. Curved, gray lines connect recombination events between *P. vivax* sequences as detected by the RDP [53] algorithm.

Next, to attempt to resolve relationships among PCR-RFLP haplotypes and geographically dispersed isolates, a pairwise distance matrix of the 48 complete sequences was constructed so as to identify pairs of more similar sequences. Figure 4A shows the tree constructed from the pairwise distance matrix using the NJ method, rooted with *P. cynomolgi*, with isolates labeled by their geographic origin and PCR-RFLP haplotype. Few nodes had greater than 50% bootstrap support, and the topology differed in the tree constructed using Bayesian phylogenetic inference (Figure 4B). The lack of resolution in the Bayesian analysis was especially apparent, as the standard deviation of the posterior probability did not drop below 0.25 even after allowing 42 million MCMC generations. This signifies statistically robust convergence was not reached for the topology displayed [39].

However, an observation from the pairwise distance matrix and one consistent across both phylogenetic methods was that there was no discernible pattern of more similar sequences having a closer geographic origin or more similar PCR-RFLP haplotype. Although five of the Venezuelan clinical isolates that shared the same PCR-RFLP haplotype (haplotype 2.1) grouped together, other Venezuelan sequences obtained from Bolívar State were distributed throughout the tree in both the Bayesian and NJ analyses (Figure 4). Furthermore, the sequences of these clinical isolates collected in Venezuela in 2003-2004 were often most similar to isolates collected distantly in time and space. Examples include Venezuelan isolate 1060, which was most similar to an isolate collected in Brazil before 1988 and an Indonesian isolate from 1990. Another example, Venezuelan isolate 166, grouped with a Vietnamese isolate from before 1978 (Figure 4). The relationship of Venezuelan isolate 1081 to the other *P. vivax* sequences was difficult to infer, with it having the least genetic distance to two Thai clinical isolates from 2000-2001 in the NJ analysis, but situating within a large, unresolved polytomy in the Bayesian tree (Figure 4). Further, the inability to resolve the cluster of five Venezuelan clinical isolates (isolates 109, 120, 127, 164, 167, and 168) from distant strains (e.g., Panama I, Chesson from New Guinea, Thai III) in either tree (Figure 4) makes it questionable whether MSP3-alpha could be used to resolve relationships at a finer scale. For epidemiologic efforts applied at a local scale, this may preclude using phylogenetics to accurately determine the origins of isolates or migration events with MSP-3α.

**Figure 5:**
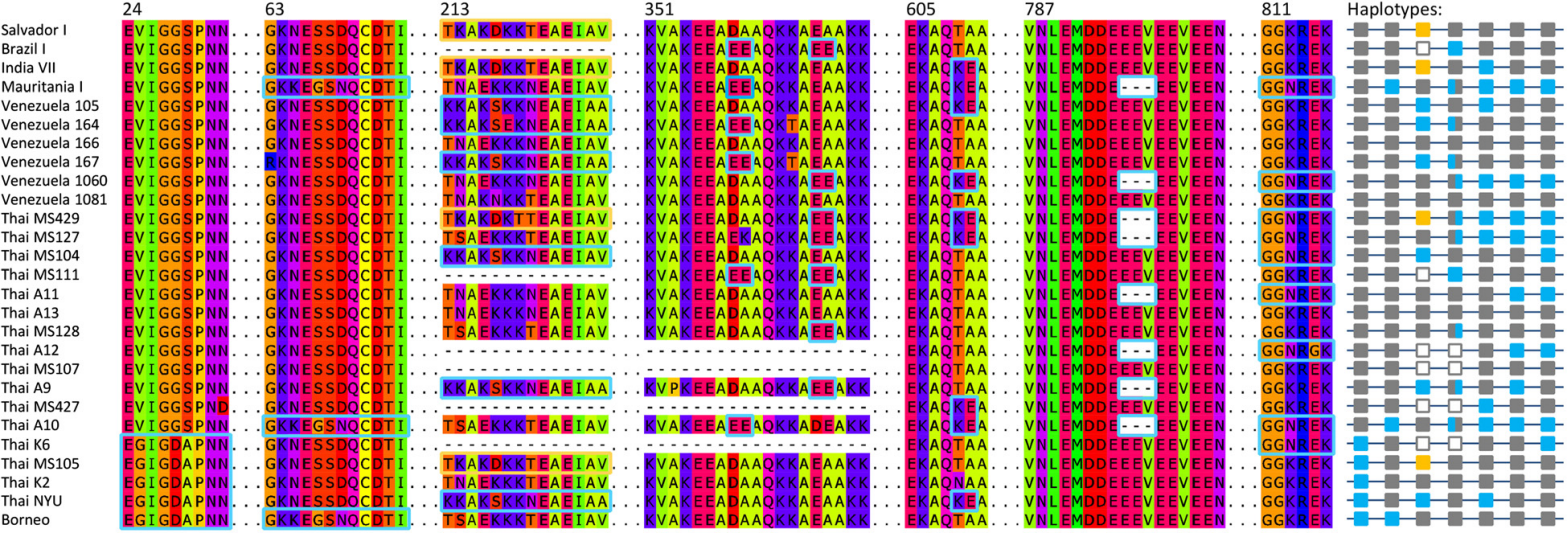
**Alignment and recombination at select MSP-3α regions.** The less frequent variants are outlined in colour for seven regions of the amino acid MSP-3α alignment (positions of the region in the full 880 residue alignment are shown above sequence blocks). Dashes represent alignment gaps due to indel events. Haplotypes are represented to the right, with the presence of a deletion (unfilled gray squares), the more common allele (filled gray square), or the rarer allele (orange or blue) marked for each of the 7 polymorphic regions. 27 of the 48 *P. vivax* sequences are shown here.

Frequent recombination at the MSP-3α locus between diverse ancestral alleles whose variation predates the migration events that led to the sample population’s current geographic distribution would be consistent with the lack of geographic patterning seen (Figure 4). To investigate this, the RDP algorithm was used to screen the alignment of *P. vivax msp-3α* sequences for recombination events. Recombination events were noticeable by eye in the alignment, where isolates that shared the same variant peptide sequence at a region with divergence in the population in one position in the alignment were often more similar to different sequences at other polymorphic regions in the alignment (Figure 5). An illustrative example is strain Mauritania I (isolated in 1998), which shared the divergent KEGSN peptide beginning at residue 65 with 5 other isolates (Figure 5). None of the Venezuelan isolates had the KEGSN motif, instead they had a sequence of NESSD. However, downstream at residue 213, two Venezuelan isolates (166 and 1060) shared the T(N/S)AEK peptide with Mauritania I and 19 other isolates, not the KKAKS sequence seen for other samples (Figure 5). Then, at residue 609, Mauritania I and in this case Venezuelan isolates 105 and 1060 had the KE motif shared by 18 isolates, while Venezuelan isolate 166 had the TA peptide shared by the other 30 of the 48 isolates (Figure 5).

This is the pattern expected by recombination shuffling the variation at these genic regions into multiple combinations, and numerous events were detected by the RDP algorithm as well. Recombination was commonly detected between isolates from different geographic regions, between isolates in different PCR-RFLP size classes, and throughout the phylogeny (Figure 4). Frequent recombination would also explain the lack of phylogenetic resolution observed and the failure for Bayesian analysis to reach convergence on an optimal topology. Indeed, Mascorro et al [16] showed previously that isolates segregated differently into phylogenies made from four different regions of *msp-3α*, indicating different segments of haplotypes have different evolutionary histories and thus yield a conflicting phylogenetic signal for the entire gene. As a result of recombination acting to shuffle genetic variation at this locus, circulating *msp-3α* haplotypes are then complex composites of multiple segments that have each likely been differentially shaped by their respective population of origin’s demography or selective environment. This seems a probable explanation for the inability to resolve relationships between distant *P. vivax* isolates, despite recovering a high number of segregating sites.

## Conclusions

*In silico* simulation of the genotyping protocol used in studies of MSP-3α diversity (see [10]) and sequence analysis of an augmented sample set detected high allelic diversity in the sample population. Whereas high diversity is considered intrinsically a desirable property in a genetic marker [10], there were incongruences observed between the patterns of nucleotide diversity at the sequence level and from the PCR-RFLP genotyping. Full length gene sequencing of small sample populations in Thailand and Venezuela failed to reveal substantial differences in nucleotide variation between these two endemic regions though they are thought to have different transmission rates and parasite population sizes [1,62-64]. Rather, local nucleotide diversity in the two small sample populations closely approximated the global *P. vivax* MSP-3α diversity from a temporally and geographically diverse sample. These findings suggest that MSP-3α nucleotide diversity is, therefore, unlikely to reflect current *P. vivax* population trends at the local scale.

While the ability to distinguish parasite haplotypes using the diverse MSP-3α locus was considered of potential use for certain molecular epidemiology applications, such as tracking recrudescence and infection clonality, this investigation finds that these PCR-RFLP haplotypes may not be informative. Specifically, it was noted that identical PCR-RFLP haplotypes were observed between very divergent sequences and that; likely due to recombination, sequences did not contain a strong signal of their evolutionary relationships. Similar patterns have been observed when studying other loci used as markers in depth such as block 2 of MSP-1 in *P. falciparum*[35], a marker that is still used in molecular epidemiologic investigations [65]. The situation could worsen in the context of mixed-cloned infections where restriction patterns are likely difficult to interpret. Thus, this study cautions against the use of these PCR-RFLP methods for comparing populations in extended geographic regions or whenever the sampling involves an extended time frame and migration is suspected.

Pilot sequencing studies are needed in order to validate whether *msp-3α* PCR-RFLP haplotypes can be tracked locally through time and space. Lack of such information hampers the ability of linking PCR-RFLP patterns with epidemiologically relevant events (e.g. a recrudescence or a re-introduction) in a given endemic area. Multi-locus microsatellite [66] or single nucleotide polymorphism panels [67] are likely more suitable for molecular epidemiologic studies than *msp-3α* PCR-RFLP or similar genotyping methods. The choice among these markers, microsatellites or SNPs, should be based on the time scale of the events that the study aims to document [66]. Overall, this study indicates that high polymorphism is a necessary but not sufficient criterion for choosing a genotyping method; it is also important to understand how such variation is generated and maintained in order to interpret the observed patterns in an epidemiological context.

## Abbreviations

MSP-3α: Merozoite surface protein-3 alpha; PCR: Polymerase chain reaction; RFLP: Restriction fragment length polymorphism; d: Overall mean genetic distance; bp: Base pair; dS: Diversity at synonymous and nonsynonymous (dN) sites; indel: Insertion-deletion mutation; MCMC: Markov Chain Monte Carlo.

## Competing interests

The authors declare that they have no competing interests.

## Authors’ contributions

AAE designed the study. MMA and MAP generated the sequencing data. BLR, MAP and AAE analysed the data. BLR wrote the initial draft of the manuscript; all authors contributed to and approved the final manuscript.

## Supplementary Material

Additional file 1 **Merozoite surface protein-3 alpha sequences analysed.** Isolates, accession codes, geographic origin, and year of isolation for the *P. vivax* sequences used in this study. Click here for file
